# A preliminary study on the mechanism of VASH2 in childhood medulloblastoma

**DOI:** 10.1038/s41598-023-42869-6

**Published:** 2023-10-11

**Authors:** Wen Liu, Yinan Fu, Meng Wang, Junhong Zhao, Julin Chen, Yongxin Wang, Hu Qin

**Affiliations:** 1 Department of Neurosurgery, The First Affiliated Hospital of Xinjiang Medical University, Urumqi, China; 2 The First Affiliated Hospital of Xinjiang Medical University, Urumqi, China; 3 Xinjiang Institute of Neurosurgery, Urumqi, China

**Keywords:** Biomarkers, Paediatric research, Cancer, Oncogenes, Tumour biomarkers, Oncology

## Abstract

To study the differences in VASH2 expression in pediatric medulloblastoma (MB) tumor tissues of different molecular subtypes, to analyze the correlation between VASH2 and the molecular subtypes of medulloblastoma, clinicopathological data, and prognosis, and to explore the specific mechanism of VASH2’s role in SHH medulloblastoma cell lines DAOY. We analyzed 47 pediatric medulloblastoma cases admitted to the Department of Pediatric Neurosurgery of the First Affiliated Hospital of Xinjiang Medical University from January 2011 to December 2019, and the expression levels of YAP1 and GAB1 in these tumor tissues were detected by immunohistochemistry (IHC) and molecularly typed (WNT-type, SHH-type, and non-WNT/SHH-type). The correlation between VASH2 and molecular typing of medulloblastoma was analyzed. We also analyzed the medulloblastoma dataset in the GEO database (GSE30074 and GSE202043) to explore the correlation between VASH2 and the prognosis of medulloblastoma patients, as well as performed a comprehensive GO enrichment analysis specifically for the VASH2 gene to reveal the underlying biological pathways of its complex molecular profile. We used vasopressin 2 (VASH2) as a research target and overexpressed and knocked down VASH2 in SHH medulloblastoma cell lines DAOY by lentiviral vectors in vitro, respectively, to investigate its role in SHH medulloblastoma cell lines DAOY cell proliferation, apoptosis, migration, invasion and biological roles in the cell cycle. (1) Among 47 pediatric medulloblastoma cases, 8 were WNT type, 29 were SHH type, and 10 were non-WNT/SHH type. the positive rate of VASH2 was highest in the SHH type with a 68.97% positive rate, followed by non-WNT/SHH and lowest in the WNT type. The results of the multifactorial analysis showed that positive expression of VASH2 was associated with medulloblastoma molecular subtype (SHH type), site of tumor development (four ventricles), and gender (male), *P* < 0.05. (2) The results of cellular experiments showed that overexpression of VASH2 increased the invasion and migration ability of medulloblast Daoy, while knockdown of VASH2 inhibited the invasion and Overexpression of VASH2 upregulated the expression of Smad2 + 3, Smad4, Mmp2 and the apoptotic indicators Bcl-2 and Caspase3, while knockdown of VASH2 suppressed the expression of Smad2 + 3 and Mmp2, and silenced the expression of Smad4 and the apoptotic indicators Bcl2, Caspase3 expression. Flow cytometric cycle analysis showed that VASH2 overexpression increased the S phase in the Daoy cell cycle, while VASH2 knockdown decreased the S phase in the SHH medulloblastoma cell lines DAOY cell cycle. Bioinformatics analysis showed that there was no statistically significant difference between the expression of VASH2 genes in the GSE30074 and GSE202043 datasets and the prognosis of the patients, but the results of this dataset analysis suggested that we need to continue to expand the sample size of the study in the future. The results of the GO enrichment analysis showed that the angiogenic pathway was the most significantly enriched, and the PPI interactions network of VASH2 was obtained from the STRING database. Using the STRING database, we obtained the PPI interaction network of VASH2, and the KEGG enrichment analysis of VASH2-related genes showed that VASH2-related genes were related to the apoptosis pathway, and therefore it was inferred that VASH2 also affects the development of tumors through apoptosis. We found for the first time that the positive expression rate of VASH2 was closely associated with SHH-type pediatric medulloblastoma and that VASH2 was involved in the invasion, migration, cell cycle, and apoptotic capacity of SHH medulloblastoma cell lines DAOY by affecting downstream indicators of the TGF-β pathway. This suggests that it is involved in the progression of pediatric medulloblastoma, and VASH2 is expected to be a diagnostic and therapeutic target for SHH-type pediatric medulloblastoma.

## Introduction

Medulloblastoma (MB) is one of the most common and malignant tumors of the central nervous system in children^1^. Long-term treatment of medulloblastoma remains a great challenge, and clinical research is currently focused on targeted therapy and immunotherapy. These novel therapies have also been gradually applied in clinical treatment, but the prognosis of children with medulloblastoma is still poor compared with the therapeutic effects of other solid tumors, and there is still a long way to go to improve the survival rate and quality of life of children with medulloblastoma^2^. This suggests that the mechanisms underlying the development of aggressive recurrence in pediatric MB are more complex, and pediatric MB patients still lack objective prognostic assessment indicators and effective therapeutic targets. Therefore, the study of its recurrence mechanism is necessary, and the exploration of its mechanism may provide ideas for the development of new therapeutic targets.

In 2021, WHO (World Health Organization) classified medulloblastoma into four molecular types: WNT-type, SHH-TP53 wild-type, SHH-TP53 mutant, and non-WNT/SHH-type. Among them, the previous group 3 and group 4 types were also categorized as non-WNT/SHH types^1^. Current clinical guidelines for individualized treatment of MB based on these four molecular typologies allow us to further improve the diagnosis of MB in children. After the diagnosis of most pediatric MB, clinicians usually adopt a combination of surgical resection and selective radiotherapy based on postoperative pathologic findings, but the overall efficacy is still unsatisfactory.

The progression mechanism of childhood MB is the result of a combination of factors, and aggressive tumor recurrence is usually the result of abnormal activation of certain signaling pathways during disease progression. Therefore, it is of great significance to study the genes involved in the progression of childhood MB recurrence and elucidate their mechanisms of action, to find specific target drugs and prognostic molecular markers for the clinical treatment of childhood MB. Our analysis of the latest findings revealed that tumor recurrence is related to the biological processes of tumor cell proliferation, invasion, and apoptosis, and is also closely related to the treatment and prognostic assessment of the disease^3^. Importantly, the main reason for the poor outcome of tumor treatment is the abnormal formation of tumor blood vessels^4^.

Antiangiogenic therapy has emerged as a potential strategy for the treatment of pediatric medulloblastoma. Angiogenesis is one of the hallmarks of tumor progression. Angiogenesis is a dynamic process and neovascularization is usually regulated by both stimulatory and inhibitory factors. However, in the pathological state of a tumor, this “angiogenic balance” may become imbalanced; dysregulation or overproduction of angiogenesis-inducing factors often leads to excessive formation of abnormal blood vessels. Recent studies have found that endogenous angiogenesis inhibitors produced by endothelial cells can regulate angiogenesis through both positive and negative feedback, and VASH2 (Vasohibin-2) belongs to this group of regulators^5^.

The family of angiogenesis inhibitors includes VASH1 (Vasohibin-1) and VASH2, where VASH1 is a negative regulator of angiogenesis and inhibits angiogenesis; VASH2, a homolog of VASH1, acts in the opposite direction of VASH1 and promotes angiogenesis during repair of damage in the body^6^. Researchers have found that VASH2 promotes the progression of solid tumors such as pancreatic and ovarian cancers through the process of epithelial–mesenchymal transition (EMT, which refers to an important underlying mechanism of embryonic development, trauma healing, and fibrotic diseases)^7^. Gene expression analyses have shown that VASH2 is expressed in endothelial cells during human and mouse embryonic development, and these expressions disappear after birth^8^. VASH2 is expressed in monocytes, and monocyte infiltration occurs at the anterior end of neovascular sprouting and promotes angiogenesis. It has been shown that VASH2 is abnormally expressed in solid tumor cells such as gastric cancer, hepatocellular carcinoma, and ovarian plasmacytoid malignancies^9^ and that VASH2 plays an important role in the process of carcinogenesis.

It is well known that childhood medulloblastoma is a tumor of embryonic origin, and medulloblastoma originates from embryonic stem cells that differentiate into neural and supportive cells. However, in some cases, the normal differentiation process of these cells is interfered with, causing them to proliferate abnormally and form tumors. The same relationship exists between the VASH2 gene and the embryo. VASH2 is a protein encoded by the human gene VASH2. It plays an important role in embryonic development and angiogenesis. Studies have shown that the VASH2 protein plays a key regulatory role in the early stages of embryonic development. It is involved in embryonic angiogenesis and the development of the vascular system. Specifically, the VASH2 protein is expressed in vascular endothelial cells and is involved in the regulation of angiogenesis and vascular density. It regulates the process of angiogenesis by inhibiting the action of vascular endothelial growth factors, such as VEGF.

In addition, the VASH2 protein has been implicated in the development of the nervous system during embryonic development. It has been found that the expression of the VASH2 protein plays an important role in regulating the nerve growth cone and neuronal migration in the embryo. It can affect neuronal growth and differentiation and is involved in the formation and connectivity of the nervous system. In other studies, it has been shown that VASH2 promotes tumor growth to a certain extent during tumor progression, but since tumor progression is a complex process, VASH2 it' s promoting role deserves to be studied in depth, based on the above research background we chose VASH2 and medulloblastoma as a research object.

## Materials and methods

### Clinical specimens

In this study, we collected paraffin specimens of 47 pediatric medulloblastoma tissues treated in the Department of Pediatric Neurosurgery of the First Affiliated Hospital of Xinjiang Medical University from January 2011 to December 2019, approved by the ethics committee of the First Affiliated Hospital of Xinjiang Medical University (ethics approval number K202111-10), and all children were not treated with radiotherapy, chemotherapy, or immunotherapy before surgery, with a postoperative follow-up of 40 The follow-up period after surgery was 40–65 months. The study complied with the 2013 revised Declaration of Helsinki and written informed consent was obtained from all patients.

### Cell lines

SHH medulloblastoma cell lines DAOY were purchased from the cell bank of the Chinese Academy of Sciences and preserved and cultured in the cell laboratory of the Clinical Research Institute of Xinjiang Medical University.

### Main reagents and instruments

VASH2 antibody (PA5-113065) was purchased from Thermo Fisher, USA. GAB1 antibody (ab59362), YAP1 antibody (ab114862), Smad4 antibody (ab230815), Smad2 + 3 antibody (ab207447), Bcl-2 antibody (ab117115), Caspase3 antibody (ab2302), β-actin antibody (ab8227), purchased from Abcam Inc, USA. Fetal bovine serum FBS, RPMI1640 basal medium, trypsin, and mycin-streptomycin double antibodies Gibco, USA, RPMI1640 medium purchased from Gibco, USA. AEC enzyme substrate kit and crystalline violet powder were purchased from Zhongshan JinQiao, China. Cell counting kit 8 purchased from Bioss Beijing, China. Transwell chambers and substrate gel (356234) were purchased from Corning, USA. MMP-2 antibody (bs-4605R) was purchased from Bosun, China. VASH2 overexpression lentivirus (42448-2), negative control virus Con238, and VASH2 knockdown lentivirus (1023B4-1), Target Seq: GCTCCAGGCGATCCAGAATTA. Negative control virus LVCON313 was purchased from Genechem, China. Purimycin solution (A610593) and BSA (A602440-0050) were purchased from Shanghai Biotech, China.

### Cell line culture and lentiviral transfection grouping

SHH medulloblastoma cell-lines DAOY were cultured in RPMI 1640 medium containing 10% fetal bovine serum at 37 °C in a 5% CO_2_ cell culture incubator, and the cells were passaged when the cell fusion reached about 85%, and SHH medulloblastoma cell-lines DAOY were transfected with lentiviral vectors provided by Genechem. medulloblastoma cell lines DAOY.

The cells were divided into a control group: NC group, VASH2 overexpression: VASH2-OE group, and VASH2 knockdown group: VASH2-si group. (where knockdown was designed for 2 targets, VASH2-si1#, and VASH2-si2#). After 48 h of lentiviral transfection, the lentiviral transfection effect was observed using fluorescence microscopy. After confirming the cell transfection efficiency, the medium was changed to a complete medium. After that, the transfected cells were screened for the stable strain with 2 μg/mL puromycin. Subsequently, control cell lines with stable growth, VASH2 overexpressing medulloblast cell lines, and VASH2 knockdown medulloblast cell lines were obtained.

### Immunohistochemistry

All paraffin sections were preheated for 0.6 h at 56 °C, dewaxed in xylene, and rehydrated by graded alcohol. Antigens were recovered by heating in citrate buffer (pH 6.0) for 20 min and quenched in a methanol and hydrogen peroxide bath for 30 min to quench endogenous peroxidase activity. Samples were then incubated overnight at 4 °C with mouse antibodies and biotinylated goat anti-mouse or rabbit IgG antibodies labeled with streptavidin-horseradish peroxidase (HRP) using a AEC staining kit according to the manufacturer's instructions. Five representative high-magnification fields (400 × magnification) are selected for each tissue section for histological evaluation. For each protein, two parameters, positive rate (PR) and staining intensity (SI) were used to describe the range and intensity of expression based on the positively stained cells in the sample. The scoring criteria were: positive staining for GAB1 and YAP1, and VASH2 showed only cytoplasmic staining with dark red granules. According to the specific detailed antibody instructions, positive and negative control, experimental sections were set up. Each pathological section was randomly selected by two pathologists in 10 fields of view under high magnification, and the results were judged by two scoring indexes, including the percentage of positive cells and staining intensity, in addition to the determination of target protein localization. Staining intensity was scored: 0 for no staining, 1 for light red, and 2 for dark red; the percentage of positively expressing cells was scored: 0 for ≤ 5%, 1 for 6–25%, 2 for 26–50%, and 3 for more than 50%. The 2 scores were multiplied and 0–2 was considered negative and greater than or equal to 3 was considered positive.

### Protein blotting analysis

Cells were harvested and washed twice with PBS (pH 7.4, 0.15 M), and total protein was extracted with RIPA buffer (Beyotime, Shanghai, China). Approximately 30 µg of total protein was subjected to SDS-PAGE and transferred to PVDF membranes, which were then blocked with 5% skim milk in TBST and incubated with primary antibody (1:1000) containing 5% BSA overnight at 4 °C. The membranes were washed three times with TBST and incubated with HRP-coupled secondary antibody (1:4000; Santa Cruz, Dallas, TX, USA) for 2 h at room temperature using enhanced chemiluminescence reagents (Chemicon International, USA) exposure and then re-probed-with-anti-β-actin-antibody (Santa Cruz, Dallas, TX, USA) at a dilution of 1:2000 to confirm the same protein loading. Detection of VASH2, Smad4, Smad2 + 3, Mmp2, Bcl-2, Caspase3.

### Cell counting kit 8 (CCK-8) assay

The transfected groups of cells (8 × 10^3^) were inoculated into 96-well plates and incubated with CCK-8 solution (10 µL; Bioss, Beijing, China) at different periods (2, 24, 48, 72 h) and 37 °C for 2 h. Cell viability was expressed by absorbance measured at 450 nm.

### Transwell assay

Matrigel and Transwell were used to perform SHH medulloblastoma cell lines DAOY invasion assays to construct invasion chambers for the separation of highly invasive and less invasive cells. Cells were inoculated in Matrigel at a density of 1 × 10^5^ cells and 100-μL serum-free RPMI-1640 in a 24-well plate Transwell system (Corning, NY, USA) with an 8-μm pore size polycarbonate filter membrane. The lower chamber contains a medium containing 10% FBS. Cells were incubated for 48 h. Cells on the lower surface of the membrane were then fixed in methanol and stained with 1% crystal violet. The stained membranes were photographed by microscopy and the invading cells were counted. The experiment was repeated at least 3 times.

### Flow cytometry

The concentration of antibody used for staining and the co-incubation time were calculated according to the manufacturer’s instructions. 12.5 µg/mL of PE anti-human flatfoot protein antibody was used. 1 × 10^5^ cells were resuspended in 100 µL PBS before washing and fixation with 4% paraformaldehyde. no cells were permeabilized because all protein markers were membrane proteins. Samples were analyzed by BD FACS Aria flow cytometry (BD Biosciences).

### Bioinformatics analysis

Obtain the GSE30074 and GSE202043 datasets (medulloblastoma samples) and their corresponding clinical information from the GEO website. Precisely aligned gene expression matrices and added annotations to genes from the platform annotation files to reveal the expression levels of the VASH2 gene in the medulloblastoma domain. Rigorously collect clinical data, exclude samples lacking survival data, and harmoniously integrate survival statistics with VASH2 gene expression profiles. Patients were expertly divided into two distinct cohorts based on the median expression level of the enigmatic VASH2 gene and analyzed in depth by the highly respected Kaplan–Meier method, presenting the data in a beautiful KM curve. At the same time, a comprehensive GO enrichment analysis was performed specifically for the VASH2 gene, revealing the underlying biological pathways of its intricate molecular pedigree. R software (https://www.r-project.org) was used for statistical analysis and mapping. The STRING database was utilized to obtain the PPI interactions network of VASH2, and the VASH2-associated genes were enriched and analyzed.

### Statistical analysis

Data were analyzed using SPSS 17.0. Quantitative data were expressed as mean ± standard deviation, and Student's *t*-test was used to analyze statistical comparisons between the two groups. For more than two groups, one-way ANOVA was used to assess differences between all groups, and then the least significant difference (LSD) method was used to compare differences between the two groups. Statistical significance was set at *P* < 0.05.

### Statement of ethics

This study was approved by the Ethics Committee of Xinjiang Medical University (ethical approval number K202111-10), and written informed consent was obtained from the patients.

## Results

### Molecular typing of medulloblastoma by immunohistochemical results

**Figure 1:**
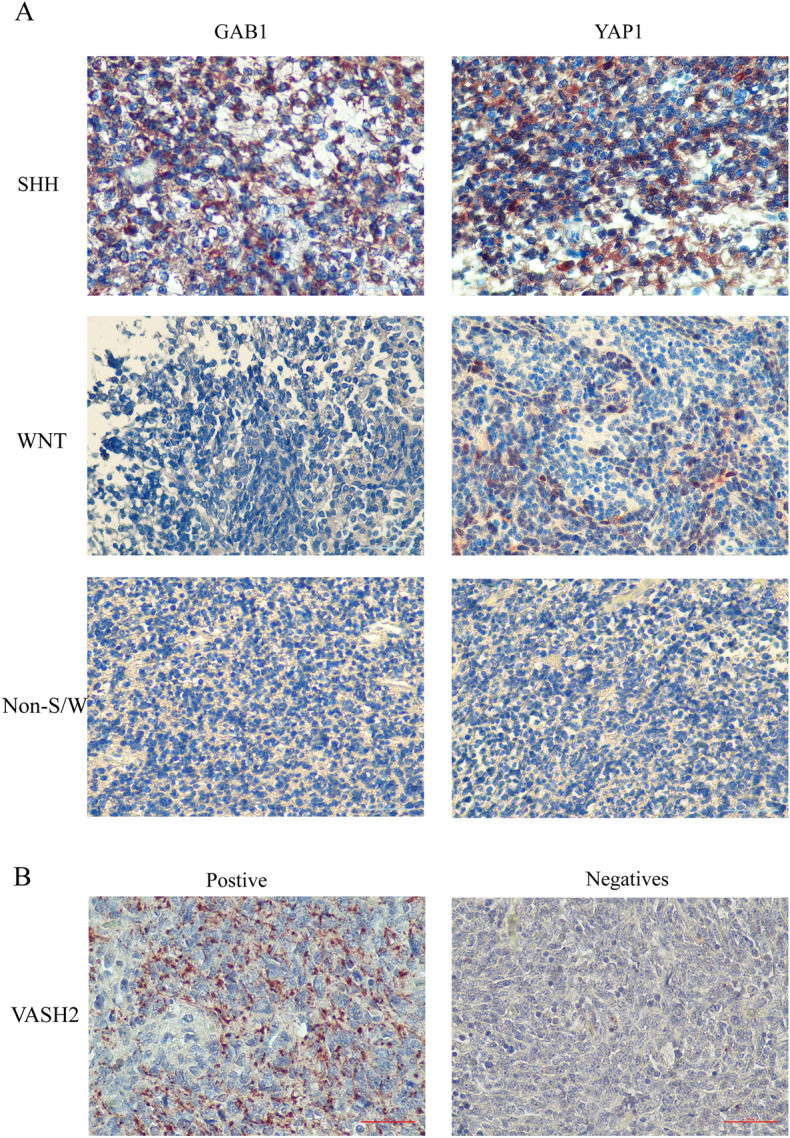
Expression of VASH2 in MB molecular subtype cohort based on IHC assay. (**A**) Molecular typing validation of MB cohort based on the IHC method. (**B**) Representative immunohistochemical staining of VASH2 in MB tumor tissue.

Molecular typing of medulloblastoma tissues was performed according to immunohistochemistry, and the results were based on the positivity of GAB1 and YAP1 as the typing, with both GAB1 and YAP1 positive as SHH type, GAB1 negative and YAP1 positive as WNT type, and both GAB1 and YAP1 negative as non-WNT/SHH type. Of the final 47 patients, 8 were WNT, 29 were SHH, and 10 were non-WNT/SHH. See Fig. 1.

### Expression of VASH2 in various types of childhood medulloblastoma

VASH2 was expressed to varying degrees in the tissues of all three subtypes of childhood medulloblastoma. 25 cases of VASH2 were positive and 22 cases were negative in 47 cases of childhood medulloblastoma, a positive rate of 53.19%; VASH2 was positive in 2 cases, and negative in 6 cases of 8 cases of WNT-type medulloblastoma, a positive rate of 25%. 20 cases of VASH2 were positive and negative in 29 cases of SHH-type medulloblastoma, with a positive rate of 68.97%, and VASH2 was positive in 3 cases and negative in 7 cases of 10 cases of non-SHH/WNT-type medulloblastoma, a positive rate of 30%. VASH2 was positive in 20 cases of SHH-type medulloblastoma and negative in 9 cases, with a positive rate of 68.97%, and VASH2 was positive in 3 cases of non-SHH/WNT-type medulloblastoma and negative in 7 cases, with a positive rate of 30%. lowest. See Table 1.

**Table 1:** Expression of VASH2 in various types of childhood medulloblastoma.

	Positive	Negative	*χ*^2^	P
WNT	2	6	7.383	0.023
Non-WNT/SHH	3	7		
SHH	20	9		

### Association of positive VASH2 expression with clinicopathological features of childhood medulloblastoma

The difference in VASH2 expression in pediatric medulloblastoma tumor tissue was not statistically significant (*P* > 0.05) concerning patient non-WNT/SHH type, age, whether the tumor metastasized, survival months, survival outcome, whether radiotherapy was given, and whether chemotherapy was given; the difference in VASH2 expression in pediatric medulloblastoma tissue was statistically significant (*P* < 0.05) concerning patient SHH type, fourth ventricle, and gender (male). tumor tissues showed statistically significant differences in expression with patient SHH type, fourth ventricle, and gender (male) (*P* < 0.05). See Table 2.

**Table 2:** Correlation of VASH2 expression with clinicopathological parameters of childhood medulloblastoma.

	β	SE	Wald	OR (95% CI)	P-value
Non-WNT/SHH	0.182	1.516	0.014	1.200 (0.061, 23.410)	0.904
SHH	3.308	1.474	5.039	27.321 (1.521, 490.686)	0.025
Age	0.073	0.124	0.344	1.075 (0.843, 1.371)	0.557
Fourth ventricle	− 3.118	1.332	5.480	0.044 (0.003, 0.602)	0.019
Gender (male)	2.004	1.006	3.969	7.418 (1.033, 53.269)	0.046
Transfers	0.930	1.238	0.565	2.536 (0.224, 28.713)	0.452
Survival month	− 0.043	0.026	2.712	0.958 (0.910, 1.008)	0.100
Survival ending	− 0.714	1.369	0.272	0.490 (0.033, 7.167)	0.602
Radiotherapy	− 0.289	1.471	0.039	0.749 (0.042, 13.380)	0.844
Chemotherapy	2.569	1.426	3.245	13.048 (0.798, 213.400)	0.072

### Relationship between VASH2 expression and prognosis of children with pediatric medulloblastoma

**Figure 2:**
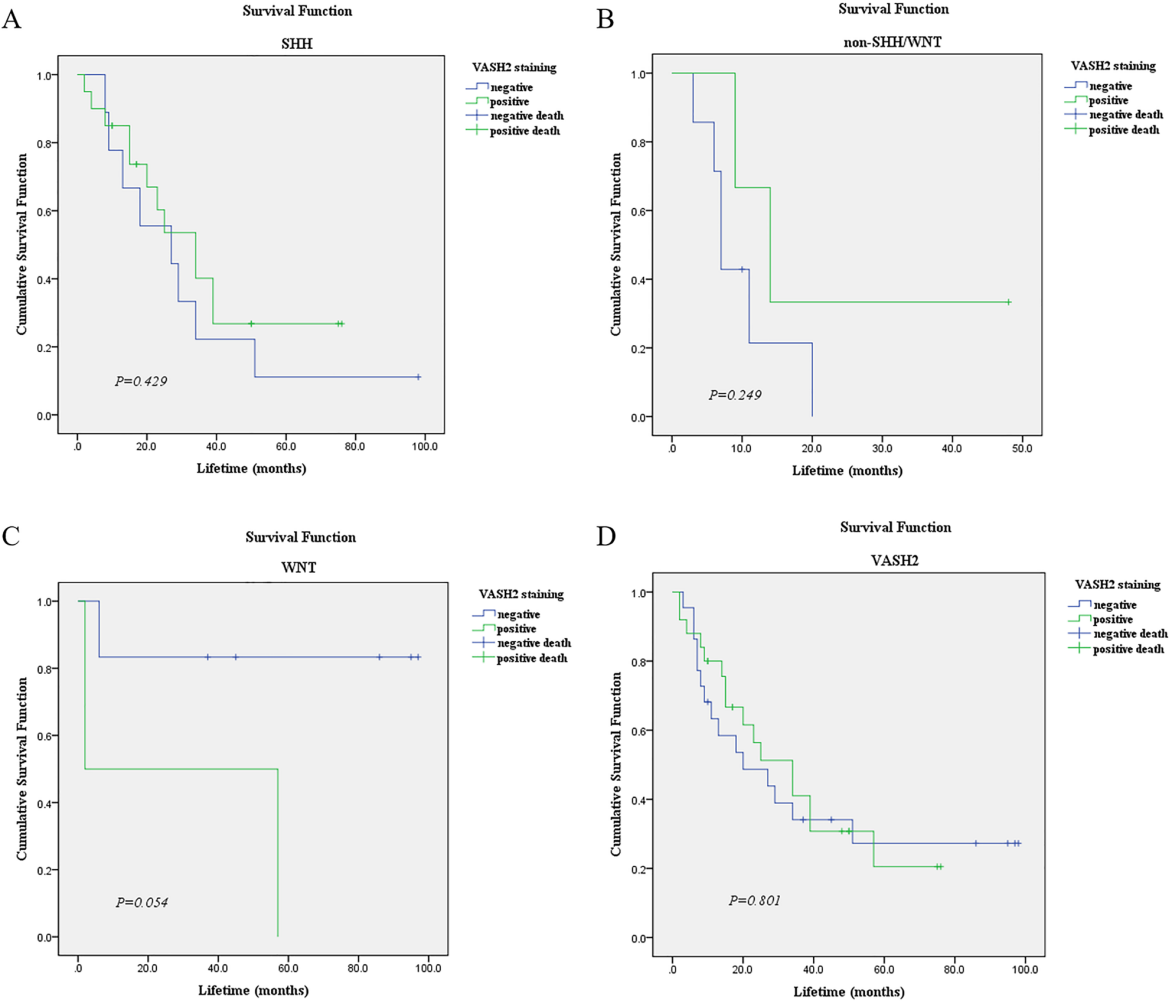
Relationship between VASH2 expression and prognosis of children with pediatric medulloblastoma. (**A**) Relationship between VASH2 and survival of children with SHH-type pediatric medulloblastoma. (**B**) Relationship between VASH2 and survival of children with non-SHH/WNT-type pediatric medulloblastoma. (**C**) Relationship between VASH2 and survival of children with WNT-type pediatric medulloblastoma. (**D**) Relationship between VASH2 and survival of children with pediatric followoblastoma.

The protein positivity rate of VASH2 was not statistically related to the age of the children, whether metastasis occurred, months of survival, or survival outcome, *P* > 0.05. Because the number of WNT type groups was not sufficient for normality, the WNT type could not be statistically described, and the results of survival analysis showed that the expression of VASH2 was not related to the prognosis of children with the three myeloid subtypes, which may be related to the small sample size included in this study. See Fig. 2.

### Mapping of lentiviral infection conditions and construction of stable expression strains

After transfecting SHH medulloblastoma cell lines DAOY with MOI = 20 for 48 h, the strongest green fluorescence intensity was seen in the cells and the cells were in a stable growth state. The puromycin was added to the basal medium and the transfected cells were screened for stable growth cell lines with puromycin medium containing a final concentration of 2 μg/mL.

### Western blot to detect the effect of VASH2, Smad2 + 3, Smad4, Mmp2, Bcl2, Caspase3 expression levels in each group of cells

**Figure 3:**
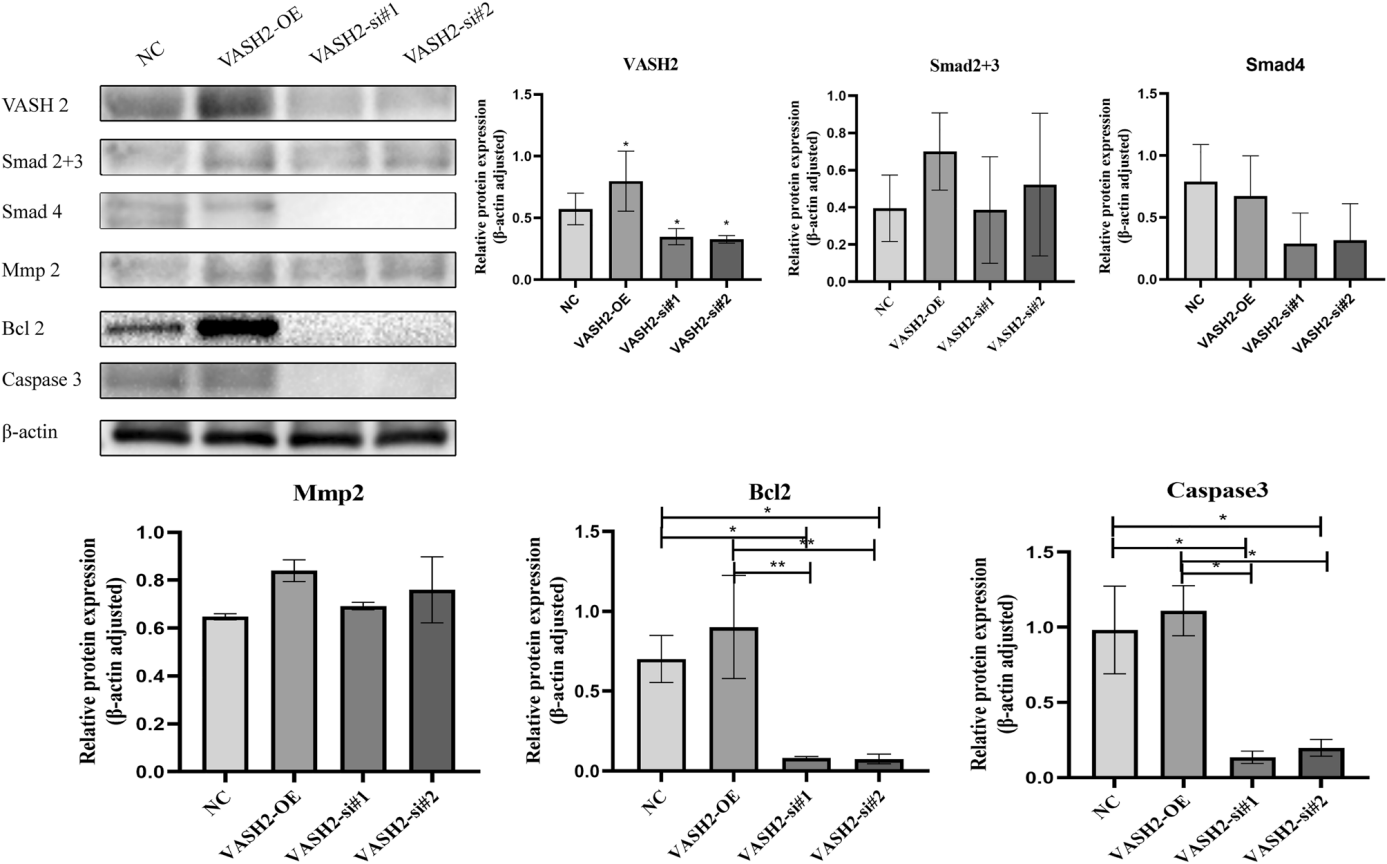
Western blot detection of VASH2, Smad2 + 3, Smad4, Mmp2, Bcl2, Caspase3 expression levels in each group of cells.

We used western blot to detect the expression of VASH2, TGFβ pathway downstream indicators Smad2 + 3, Smad4, Mmp2, and apoptosis indicators Bcl2 and Caspase3 protein levels in SHH medulloblastoma cell lines DAOY after overexpression and knockdown of VASH2, respectively, using β-actin as an internal reference. The results showed that Smad2 + 3, Smad4, Bcl2, and Caspase3 expression was changed in SHH medulloblastoma cell lines DAOY regardless of overexpression or knockdown of VASH2, indicating that VASH2 expression in myeloblasts may be mediated by affecting downstream indicators of TGFβ pathway. The expression of Bcl2 and Caspase3 in the VASH2-OE group was the most significant difference compared to the other two groups (*P* < 0.05), and the results showed that VASH2 may be a key factor affecting apoptosis in SHH medulloblastoma cell-lines DAOY. This also supports the results of the immunohistochemistry experiments in terms of molecular mechanisms. See Fig. 3. In order to improve the clarity and simplicity of the presentation, cropped blots are displayed in the main paper, original blots are presented in Source data.

### Cell migration and invasion

**Figure 4:**
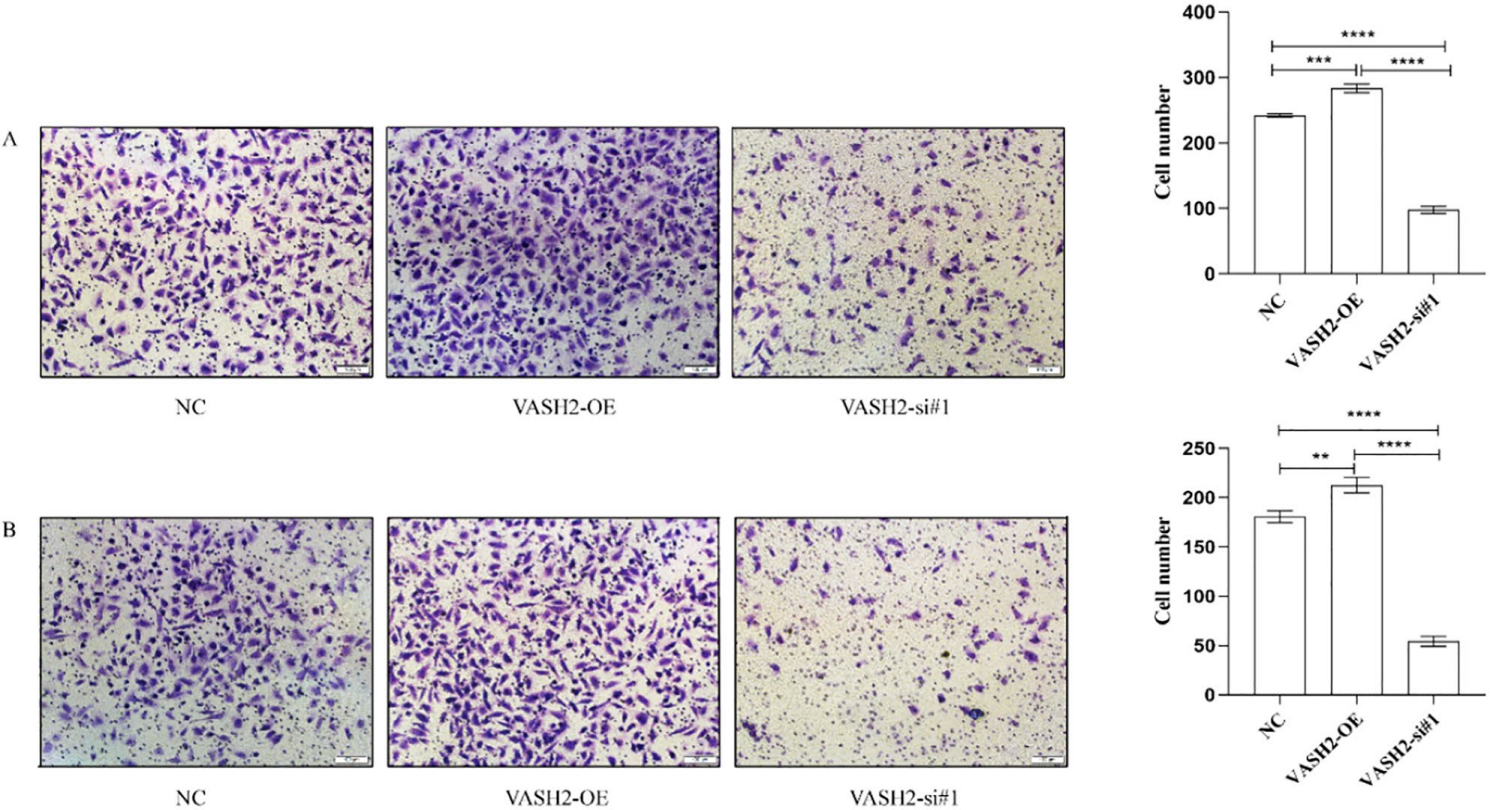
Effect of VASH2 overexpression and knockdown on Daoy invasion and migration ability of follower cells. (**A**) Effect of VASH2 overexpression and knockdown on Daoy invasion ability of medulloblast cells. (**B**) Effect of VASH2 overexpression and knockdown on Daoy migration ability of medulloblast cells.

We used Transwell chambers to detect the effects of VASH2 overexpression and VASH2 silencing on the migration and invasion ability of SHH medulloblastoma cell lines DAOY. By counting the cells in the VASH2-OE group versus the VASH2-si-RNA group and the cells in the empty NC group that penetrated the basement membrane of the Transwell. The results showed that the number of cells penetrating the basement membrane of Transwell was higher in the VASH2-OE group than in the VASH2-si-RNA and empty-loaded groups, and the difference was statistically significant (*P* < 0.05), suggesting that overexpression of VASH2-OE significantly promoted the migration and invasion ability of SHH medulloblastoma cell-lines DAOY, and VASH2-si-RNA significantly inhibited the migration and invasion ability of medulloblast migration and invasion ability of SHH medulloblastoma cell-lines DAOY, (*P* < 0.05). See Fig. 4.

### CCK-8 assay to detect the effect of VASH2 on the proliferative capacity of medulloblast Daoy

**Figure 5:**
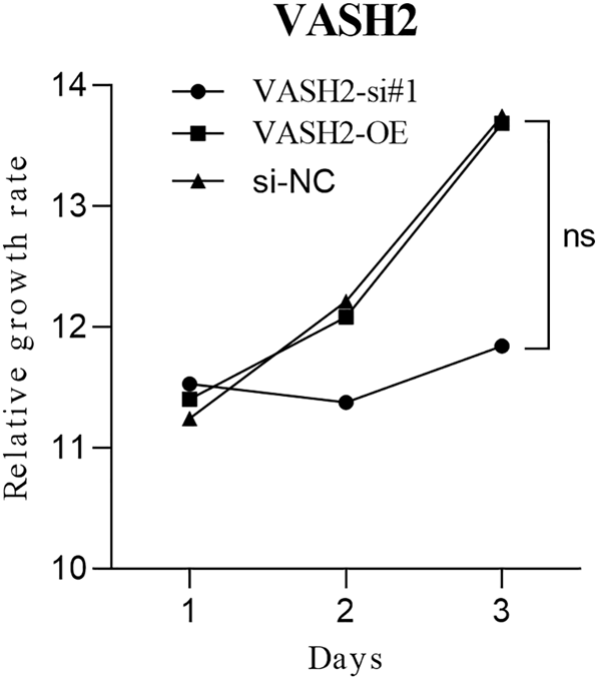
Growth curves of Daoy cells overexpressing VASH2 and knocking down VASH2.

The proliferation ability of cells in the VASH2-NC, VASH2-OE, and VASH2-si groups was examined using the CCK-8 assay (Cell Counting Kit-8). The results of the assay showed no statistical difference in the proliferation ability of the three groups, (*P* > 0.05) thus it is clear that modulation of VASH2 does not affect the proliferation ability of SHH medulloblastoma cell lines DAOY. See Fig. 5.

### Cell cycle

**Figure 6:**
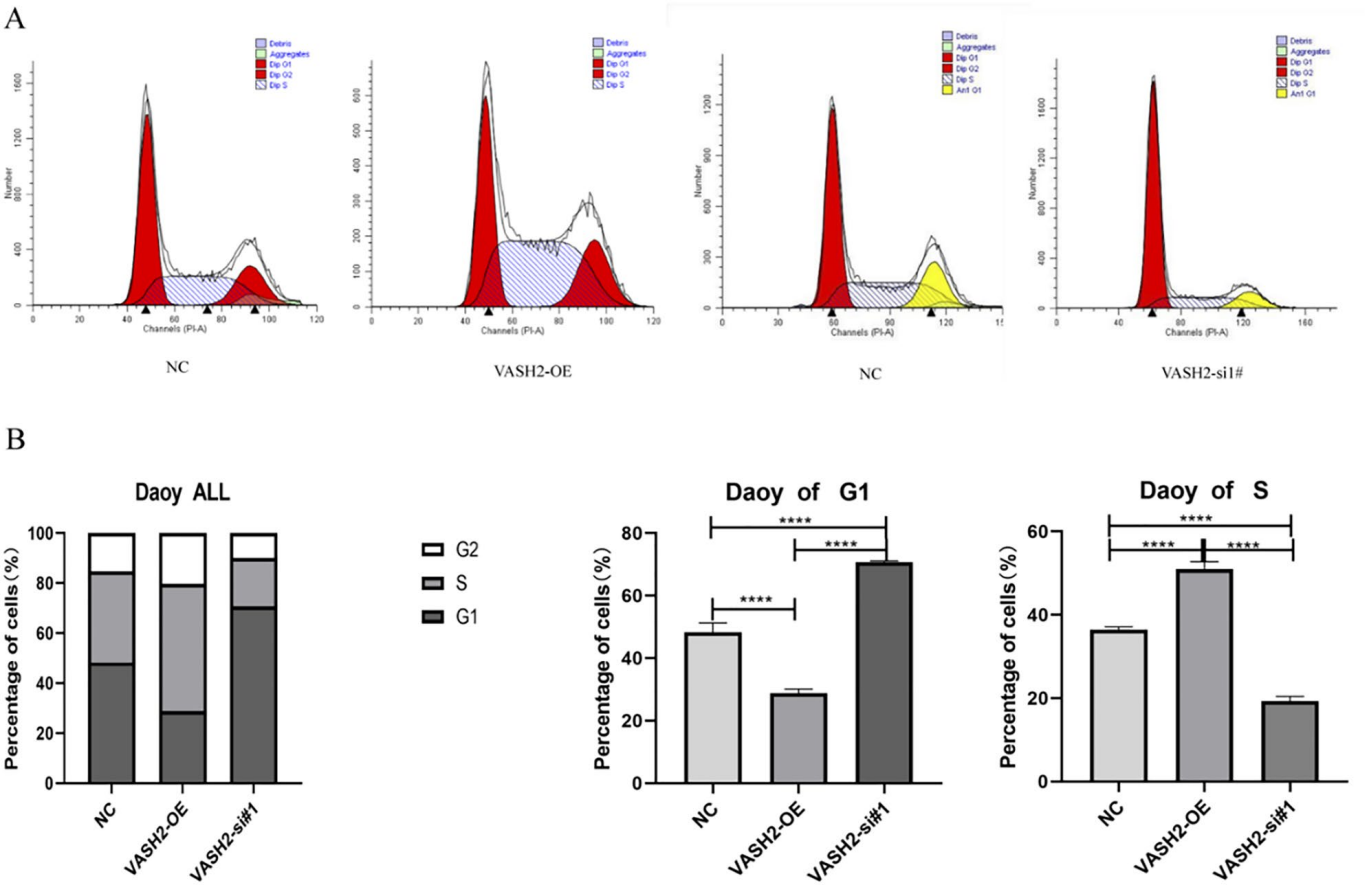
Effects of VASH2 overexpression and knockdown on Daoy G1 and S phases of medulloblast cells. (**A**) Effect of VASH2 overexpression and knockdown on Daoy cycle of medulloblast cells. (**B**) Effect of VASH2 overexpression and knockdown on G1 and S phases of medulloblast cell cycle.

Flow cytometry was used to analyze the alteration of the SHH medulloblastoma cell lines DAOY cell cycle by overexpression and silencing of the VASH2 gene. Flow cycle results showed that overexpression of VASH2 decreased the G1 phase of medulloblast cells, while knockdown of VASH2 significantly increased the G1 phase of SHH medulloblastoma cell lines DAOY, *P* < 0.05; meanwhile, the proportion of S phase of cells in the VASH2-OE group (51.57 ± 0.74%) was higher than that in the VASH2-si group (20.54 ± 1.27%) and the VASH2-NC group (35.85 ± 1.34%), and the differences were statistically significant (*P* < 0.05), i.e., relative to the VASH2-si and VASH2-NC groups, the cell cycle of the VASH2-OE group was blocked in S phase, with prolonged DNA synthesis and active cell division. The results suggest that overexpression of VASH2 in SHH medulloblastoma cell lines DAOY promotes medulloblast proliferation, and VASH2 knockdown inhibited the proliferation of myeloblasts. See Fig. 6.

### Bioinformatics analysis

**Figure 7:**
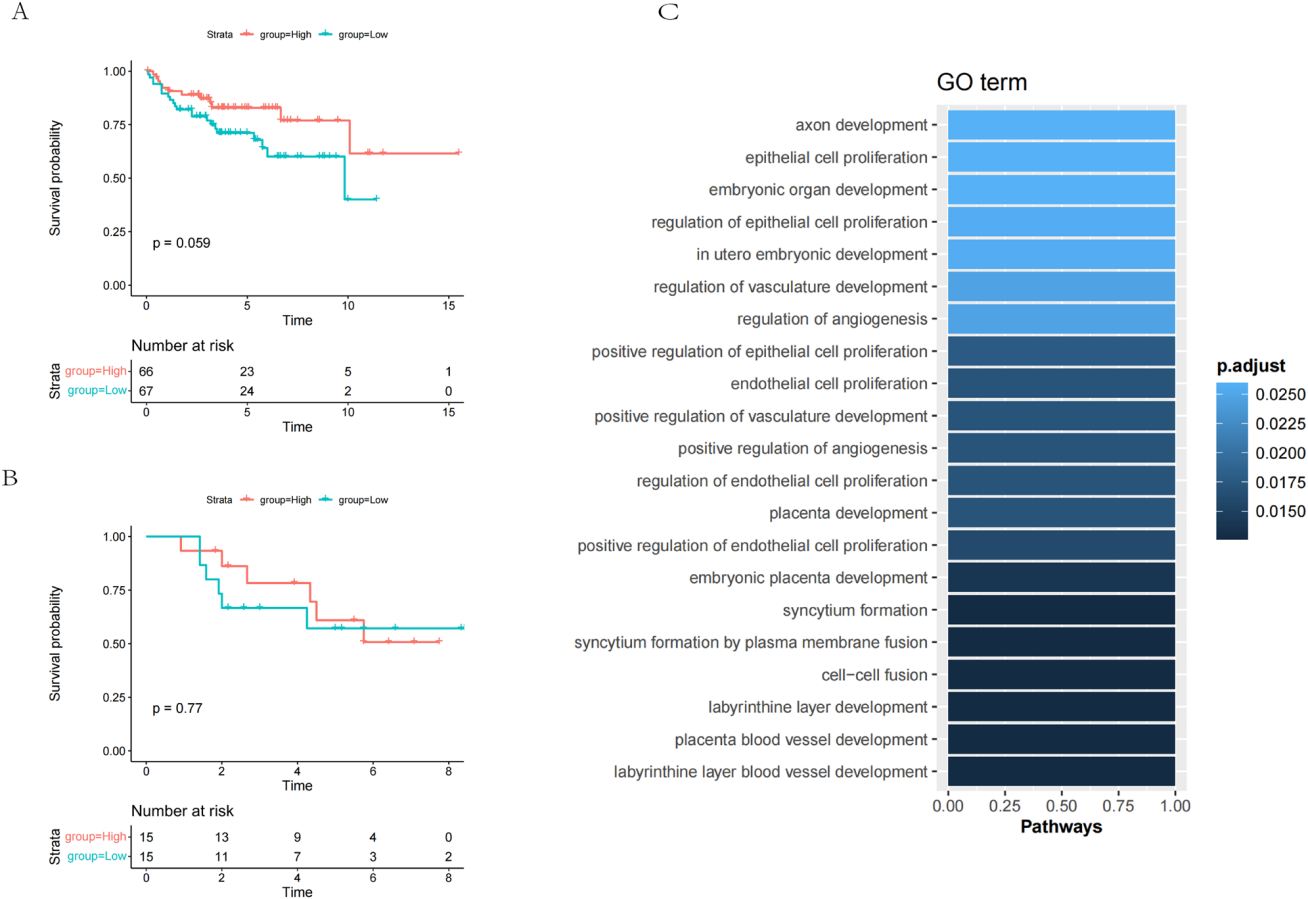
Clinical significance of VASH2 mRNA expression in human medulloblastoma. (**A**,**B**) Kaplan–Meier survival curves showing no significant correlation between survival of MB patients and VASH2 mRNA expression in two human MB databases. (**A**) GEO: GSE202043 dataset includes mRNA data from 194 medulloblastoma cases. (**B**) GEO: GSE30074 dataset including mRNA data for 30 medulloblastoma mRNA data. (**C**) GO enrichment analysis of VASH2 characteristic gene sets.

**Figure 8:**
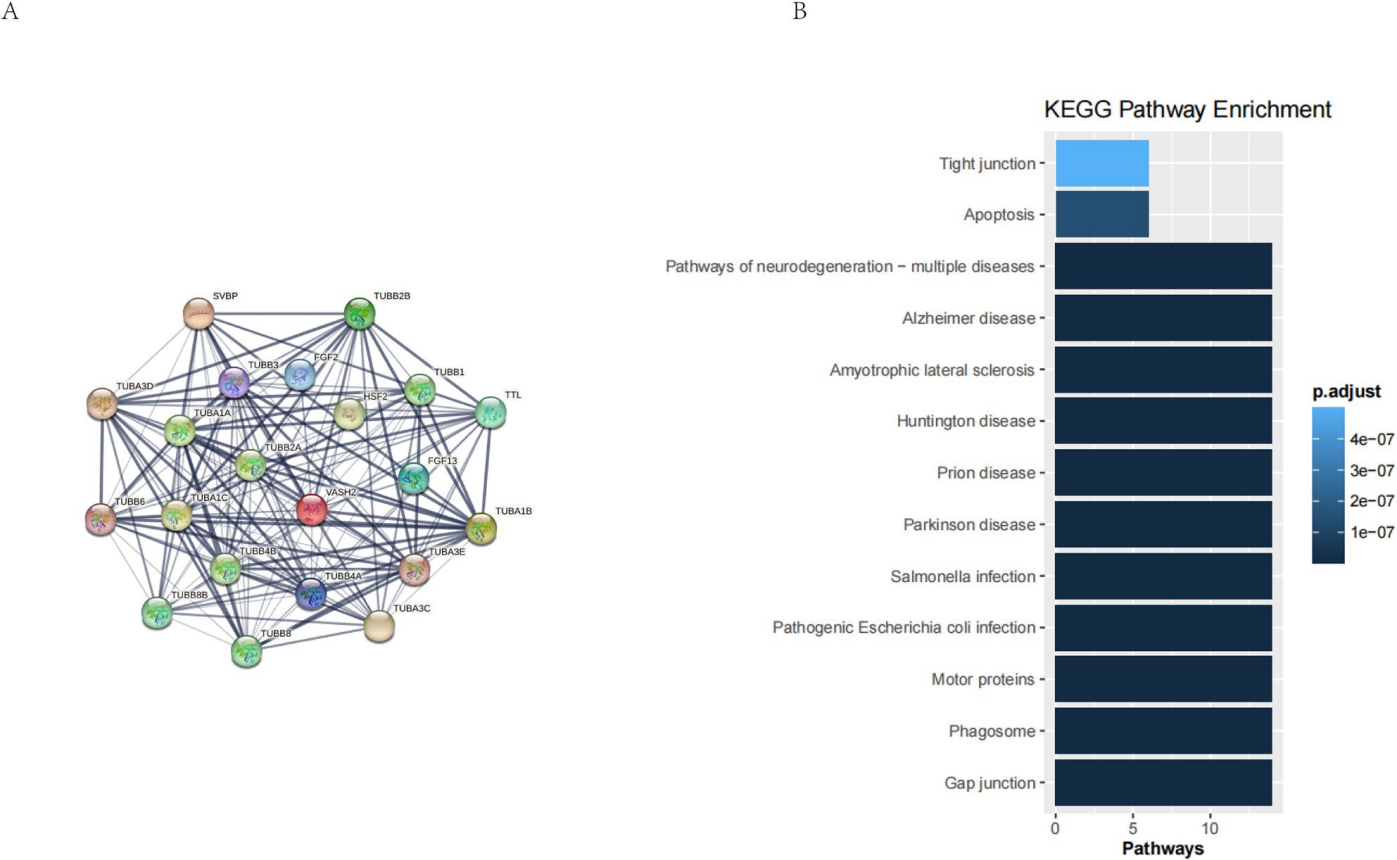
VASH2 gene characteristics and functional analysis. (**A**) String construction of VASH2 protein co-expression network. (**B**) KEGG enrichment analysis of VASH2-related signaling pathways.

Bioinformatics analysis showed that K-M survival analysis of VASH2 gene expression and patient prognosis in the GSE30074 and GSE202043 datasets still did not show any statistical difference, but the results of this dataset analysis suggest that we need to continue to expand the sample size of the study in the future^10,11^. The results of the GO enrichment analysis showed that the angiogenic pathway was the most significant, which also supports the conclusion reached in other studies of VASH2. Using the STRING database to obtain the PPI interaction network of VASH2, KEGG enrichment analysis of VASH2-related genes showed that VASH2-related genes were related to the apoptosis pathway, and therefore it was inferred that VASH2 also affects the process of tumorigenesis and development through apoptosis^12^. See Figs. 7, 8.

## Discussion

Childhood medulloblastoma is characterized by high aggressiveness, easy recurrence, and poor prognosis^13^. Even after surgical resection, radiotherapy and chemotherapy may cause a series of complications such as impaired cognitive function, which seriously affect the long-term prognosis of children and bring a heavy burden to their families and society. With the advancement of science and technology, the concept of precision and individualized treatment of tumors has been proposed, and the exploration of efficient and safe diagnostic and therapeutic target development in pediatric neuro-oncology is particularly important^14^. Since humans entered the molecular era, the application of deep sequencing technology in tumor research has enabled researchers to have a clearer understanding of tumors and more concepts of tumor molecular subtypes have been proposed, providing theoretical support for precision therapy^15^. WHO redefined pediatric medulloblastoma in 2021 into four subtypes: WNT, SHH-TP53 wild type, SHH-TP53 mutant type, and non-WNT/SHH type, incorporating the previous group3 and group4 types into the non-WNT/SHH type^1^. SHH-type medulloblastoma has been reported to have the highest incidence among patients with all types of medulloblastoma, about 1/3, and is prone to early metastasis. SHH-type medulloblastoma is often accompanied by abnormal activation of the SHH signaling pathway, and studies have shown that abnormal activation of this pathway often affects normal cerebellar development^16^. In other solid tumors, abnormal activation of the SHH pathway leads to tumorigenesis, such as cutaneous basal carcinoma and digestive tract tumors^17^. Also, abnormal expression of proteins downstream of this pathway (e.g., Smo, Gli1, Gli2, etc.) is importantly associated with the development of SHH-type medulloblastoma. The prognosis of children with SHH-type medulloblastoma varies from individual to individual, with patients with SHH-TP53 mutation having the worst prognosis, while patients with SHH-TP53 wild type have a relatively better prognosis^18^. Therefore, it is important to implement individualized and precise treatment for patients with SHH medulloblastoma and explore new targets for diagnosis and treatment. As reported earlier, the treatment of SHH-type medulloblastoma was mainly developed for inhibitors of SHH-related signaling pathway proteins, and satisfactory efficacy was achieved in previous clinical trials^19^. In the present study, children with SHH type likewise accounted for the highest proportion of cases. Currently, there are no reports on the role and mechanism of VASH2 in CNS tumors, especially in pediatric medulloblastoma, both at home and abroad; therefore, it is important to investigate the correlation between VASH2 and the pathogenesis of SHH-type medulloblastoma to enhance the prognosis of children with SHH-type medulloblastoma.

In this study, we examined the expression level of VASH2 in pediatric medulloblastoma tissues by immunohistochemistry and found that the positive expression rate of VASH2 was closely related to the molecular typing of medulloblastoma. Subsequently, we performed VASH2 regulatory modeling experiments in SHH medulloblastoma cell lines DAOY with two medulloblastoma datasets GSE30074 and GSE202043 (medulloblastoma samples), and their corresponding clinical information obtained from the GEO database. Kaplan–Meier analysis was performed to present the data as Kaplan–Meier curves. At the same time, a comprehensive GO enrichment analysis was performed specifically for the VASH2 gene, revealing the underlying biological pathways of its intricate molecular pedigree. Finally, the STRING database was utilized to obtain the PPI interactions network of VASH2, and the enrichment analysis of VASH2-associated genes was performed. The findings on the dataset partially support our conclusions and also suggest that a more comprehensive revelation of the specific mechanism of action of VASH2 in pediatric medulloblastoma in the future will require us to not only increase the sample size, but also to carry out studies in vivo, in vitro, and from medulloblastomas with different molecular typing.

The current study preliminarily elucidated the biological function of VASH2 in SHH medulloblastoma cell-lines DAOY, and conducted a preliminary exploration of its related signaling pathways, providing a theoretical basis for exploring new diagnostic and therapeutic targets for pediatric medulloblastoma. In a recent study report, researchers found that the expression level of VASH2 was abnormally elevated in some solid tumors such as gastric cancer, breast cancer, hepatocellular carcinoma, and pancreatic cancer, and promoted tumor angiogenesis under paracellular secretion, which was closely related to tumor prognosis^20,21^. One study showed that the knockdown of VASH2 significantly increased the chemosensitivity of tumor cells to paclitaxel PTX^22^. VASH2 increased the expression of ZEB1/2 in pancreatic tumors through activation of the SHH pathway, which both facilitated the EMT process and increased the resistance of pancreatic tumors to gemcitabine chemotherapy^23^. The same conclusion was reached in another study on liver tumors^24^. In addition, in a study on non-small cell lung cancer, researchers found that VASH2 promoted tumor cell proliferation and resistance to adriamycin through the AKT signaling pathway^25^. As shown in earlier studies, microtubules, as an important component of the eukaryotic cytoskeleton, are involved in a variety of cellular functions^26^. VASH2 has an atypical tyrosine-catalyzed structure that affects post-translational modification of microtubule proteins in the cell and is involved in tyrosine recycling and degradation^27^. However, dysregulation of tyrosine cycling is strongly associated with disease and cancer^28,29^. A study reported in Nature showed that microtubule-affinity-regulated kinase 4 (MARK4) regulates the contractility of cardiac myocytes by promoting the phosphorylation of microtubule-associated protein 4 (MAP4), which facilitates entry of vasodilator 2 (VASH2) into the microtubule for α-microtubule protein de-tyrosination^29^. contractility of cardiomyocytes^30^. Conversely, inhibition of microtubule deacetylation improves contractility in failing cardiomyocytes, suggesting that VASH2 is also closely related to the pathogenesis of heart failure^31,32^.

In this study, we explored the potential correlation between the high expression level of VASH2 and the pathological features of medulloblastoma (e.g., SHH subtype) using immunohistochemical techniques, and we also found that the lowest expression level of VASH2 was found in children with WNT-type medulloblastoma, suggesting that VASH2 may be a potential diagnostic target for predicting the molecular subtype of medulloblastoma. In addition, the cell cycle consists of interphase and division (M phase), and division consists of three phases: pre-DNA synthesis (G1 phase), DNA synthesis (S phase), and late DNA synthesis (G2 phase)^33^. In the G1 phase, cells synthesize RNA and proteins, whereas, in the S phase, cells mainly undergo DNA synthesis^34,35^. In the present study, we found that modulation of VASH2 overexpression in SHH medulloblastoma cell lines DAOY increased the S phase in the Daoy cell cycle, whereas knockdown of VASH2 shortened the S phase in the Daoy cell cycle. VASH2 can affect DNA synthesis in the Daoy cell cycle.

Apoptosis is a genetically controlled process of programmed cell death and is an active process under the balance of intracellular environment^36,37^. Apoptosis plays an important role in many biological developmental processes in organisms, such as the turnover of old and new cells in the body, biological embryonic development, disease, and tumor progression^38,39^. In most tumors, the unlimited proliferation of tumor cells is inextricably linked to apoptosis^40^. The results of this study showed that overexpression of VASH2 in myeloblastoma cells Daoy led to a significant increase in the expression level of Bcl2 (B-cell lymphoma/leukemia-2 gene) in myeloblastoma cells, whereas knockdown of VASH2 significantly inhibited the expression of Bcl2 in SHH medulloblastoma cell-lines DAOY. Meanwhile, the trend of caspase3 in medulloblastoma cell lines DAOY after modulation of VASH2 was consistent with that of Bcl2, a proto-oncogene that inhibits apoptosis, and earlier studies have shown that Bcl2 is a downstream target gene of the SHH signaling pathway, and the expression level of SHH in medulloblastoma cell-lines DAOY was significantly increased by knockdown of VASH2, while the expression level of Bcl2 in SHH medulloblastoma cell-lines DAOY was significantly inhibited by knockdown of VASH2. SHH signaling pathway in DAOY is in turn derived from SHH-type medulloblastoma^41,42^. Overexpression of Bcl2 in cells protects against Fas ligand-induced cell death^43^. The results of basic research have shown that Bcl2 enhances the resistance of most tumor cells to DNA damage^44^, thus improving tolerance to chemotherapeutic agents, but Bcl2 does not promote DNA self-repair^45^. p53 is also an oncogene, and mutations in this gene occur in more than half of malignant tumors and are also a molecular indicator of DNA damage, and studies have shown that Bcl2 inhibits p53-mediated apoptosis^46,47^. The results of one study showed that VASH2 could inhibit apoptosis in liver tumor cells by down-regulating p53, and earlier results showed that the p53 gene was highly expressed in SHH medulloblastoma cell lines DAOY, thus suggesting that VASH2 could also mediate apoptosis in SHH medulloblastoma cell-lines DAOY through the p53 gene^48^. Caspase3 is an important executor in the apoptotic process. A study showed that overexpression of VASH2 resulted in an increased abundance of intracellular Caspase3^24^. This finding is also consistent with the results of our current study. The above findings suggest that VASH2 can regulate the apoptotic ability in SHH medulloblastoma cell lines DAOY, and VASH2 may be an important regulator in the biological process of medulloblastoma.

One of the most important biological features of malignant medulloblastoma is its aggressive growth. Medulloblastoma cells tend to invade the surrounding normal brain tissues at an early stage, and it is difficult to distinguish them from normal brain tissues with clear boundaries. In this study, we found for the first time that the aberrant expression of VASH2 in pediatric medulloblastoma is closely related to the molecular subtype of medulloblastoma, and VASH2 regulates the invasion, migration, cell cycle, and apoptosis of medulloblastoma mainly through the TGF-β signaling pathway. This suggests that it is involved in the progression of childhood medulloblastoma and plays an important role in the process of medulloblastoma development and progression, and could be a target for medulloblastoma diagnosis and treatment.

### Limitations

There are some limitations of this study. Due to the large number of lost cases, the final sample size included in this study was 47 cases, and exploring the prognostic relationship between VASH2 and medulloblastoma still needs to be investigated in a large sample cohort in the future. In addition, the current gold standard for molecular typing of medulloblastoma mainly relies on DNA methylation assay^49^, but this method is expensive and has special requirements for the preservation and transportation of tumor tissues, therefore, in this study, we adopted a cost-effective immunohistochemistry method to molecularly type medulloblastoma, and our experiments were mainly conducted using SHH medulloblastoma cell-lines DAOY for the study, this cell line is currently a common model used internationally to study gene function in medulloblastoma and is widely used in many medulloblastoma-related studies. In addition, we used a variety of experimental methods to ensure the reliability and reproducibility of our findings. Although we initially realized that the use of a single cell line might limit the depth and breadth of our study, and we were unable to obtain other cell lines suitable for our study under the current circumstances, we will try to use other medulloblastoma cell lines for simultaneous validation in our future studies, and we will also carry out experiments at the animal level for the experimental studies to further strengthen our findings.

## Conclusion

We found for the first time that the positive expression rate of VASH2 was closely associated with SHH-type pediatric medulloblastoma and that VASH2 was involved in the invasive, migratory, cell cycle, and apoptotic abilities of SHH medulloblastoma cell lines DAOY by affecting downstream indicators of the TGF-β pathway.VASH2 is expected to be a diagnostic and therapeutic target.

### Supplementary Information

Supplementary Information.

## Data Availability

Data supporting the results of this study are available in the paper and in the Supplementary Information. Additional information can be obtained from first author Wen Liu. The datasets analysed during the current study are available in the [GEO] repository, [GSE30074 and GSE202043].

## References

[1] Louis DN, Arie P, Pieter W (2021). The 2021 WHO classification of tumors of the central nervous system: A summary. Neuro Oncol..

[2] Menyhárt O, Győrffy B (2020). Molecular stratifications, biomarker candidates and new therapeutic options in current medulloblastoma treatment approach. Cancer Metastasis Rev..

[3] Sikkema AH (2012). EphB2 activity plays a pivotal role in pediatric medulloblastoma cell adhesion and invasion. Neuro Oncol..

[4] Zheng R, Li F, Li F (2021). Targeting tumor vascularization: Promising strategies for vascular normalization. J. Cancer Res. Clin. Oncol..

[5] Sato Y (2012). The vasohibin family: Novel regulators of angiogenesis. Vascul. Pharmacol..

[6] Yasufumi S (2013). The vasohibin family: A novel family for angiogenesis regulation. J. Biochem..

[7] Norita R (2017). Vasohibin-2 is required for epithelial–mesenchymal transition of ovarian cancer cells by modulating transforming growth factor-β signaling. Cancer Sci..

[8] Ma D, Wu L, Li S (2017). Vasohibin2 promotes adriamycin resistance of breast cancer cells through regulating ABCG2 via AKT signaling pathway. Mol. Med. Rep..

[9] Suzuki Y (2017). Requisite role of vasohibin-2 in spontaneous gastric cancer formation and accumulation of cancer-associated fibroblasts. Cancer Sci..

[10] Park AK, Lee SJ, Phi JH, Wang KC (2012). Prognostic classification of pediatric medulloblastoma based on chromosome 17p loss, expression of MYCC and MYCN, and Wnt pathway activation. Neuro Oncol..

[11] Cho YJ, Tsherniak A, Tamayo P, Santagata S (2011). Integrative genomic analysis of medulloblastoma identifies a molecular subgroup that drives poor clinical outcome. J. Clin. Oncol..

[12] Kanehisa M, Furumichi M, Sato Y, Kawashima M, Ishiguro-Watanabe M (2023). KEGG for taxonomy-based analysis of pathways and genomes. Nucleic Acids Res..

[13] Li BK, Al-Karmi S, Huang A (2020). Pediatric embryonal brain tumors in the molecular era. Expert Rev. Mol. Diagn..

[14] Wang SS, Bandopadhayay P, Jenkins MR (2019). Towards immunotherapy for pediatric brain tumors. Trends Immunol..

[15] Cobain EF, Wu Y-M, Vats P (2021). Assessment of clinical benefit of integrative genomic profiling in advanced solid tumors. JAMA Oncol..

[16] Guo D (2021). Tumor cells generate astrocyte-like cells that contribute to SHH-driven medulloblastoma relapse. J. Exp. Med..

[17] Jiang J (2022). Hedgehog signaling mechanism and role in cancer. Semin. Cancer Biol..

[18] Menyhárt O, Győrffy B (2019). Principles of tumorigenesis and emerging molecular drivers of SHH-activated medulloblastomas. Ann. Clin. Transl. Neurol..

[19] Wei Y, Maximov V, Morrissy SA (2019). p53 function is compromised by inhibitor 2 of phosphatase 2A in sonic hedgehog medulloblastoma. Mol. Cancer Res..

[20] Xue X, Zhang Y, Zhi Q (2014). MiR200-upregulated Vasohibin 2 promotes the malignant transformation of tumors by inducing epithelial–mesenchymal transition in hepatocellular carcinoma. Cell Commun. Signal..

[21] Inoue C, Miki Y, Saito-Koyama R (2022). Vasohibin-1 and -2 in pulmonary lymphangioleiomyomatosis (LAM) cells associated with angiogenic and prognostic factors. Pathol. Res. Pract..

[22] Koyanagi T, Saga Y, Takahashi Y (2021). Knockout of vasohibin-2 reduces tubulin carboxypeptidase activity and increases paclitaxel sensitivity in ovarian cancer. Cancer Med..

[23] Zhang Y, Xue X, Zhao X (2018). Vasohibin 2 promotes malignant behaviors of pancreatic cancer cells by inducing epithelial–mesenchymal transition via Hedgehog signaling pathway. Cancer Med..

[24] Li Z, Tu M, Han B (2014). Vasohibin 2 decreases the cisplatin sensitivity of hepatocarcinoma cell line by downregulating p53. PLoS One.

[25] Tan X, Liao Z, Zou S (2020). VASH2 promotes cell proliferation and resistance to doxorubicin in non-small cell lung cancer via AKT signaling. Oncol. Res..

[26] Goodson HV, Jonasson EM (2018). Microtubules and microtubule-associated proteins. Cold Spring Harb. Perspect. Biol..

[27] Nieuwenhuis J, Adamopoulos A, Bleijerveld OB (2017). Vasohibins encode tubulin detyrosinating activity. Science.

[28] Magiera MM, Singh P, Gadadhar S (2018). Tubulin posttranslational modifications and emerging links to human disease. Cell.

[29] Nieuwenhuis J, Brummelkamp TR (2019). The tubulin detyrosination cycle: Function and enzymes. Trends Cell Biol..

[30] Yu X, Chen X, Amrute-Nayak M (2021). MARK4 controls ischaemic heart failure through microtubule detyrosination. Nature.

[31] Arce CA, Barra HS (1985). Release of C-terminal tyrosine from tubulin and microtubules at steady state. Biochem. J..

[32] Shigematsu H, Imasaki T, Doki C (2018). Structural insight into microtubule stabilization and kinesin inhibition by Tau family MAPs. J. Cell Biol..

[33] Dalton S (2015). Linking the cell cycle to cell fate decisions. Trends Cell Biol..

[34] Bertoli C, Skotheim JM, de Bruin RAM (2013). Control of cell cycle transcription during G1 and S phases. Nat. Rev. Mol. Cell Biol..

[35] Takeda DY, Dutta A (2005). DNA replication and progression through S phase. Oncogene.

[36] Xu X, Lai Y, Hua ZC (2019). Apoptosis and apoptotic body: Disease message and therapeutic target potentials. Biosci. Rep..

[37] Cheng X, Ferrell JE (2018). Apoptosis propagates through the cytoplasm as trigger waves. Science.

[38] Takuma K, Baba A, Matsuda T (2004). Astrocyte apoptosis: Implications for neuroprotection. Prog. Neurobiol..

[39] Thompson EB (1998). Special topic: Apoptosis. Annu. Rev. Physiol..

[40] Fujiwara C, Muramatsu Y, Nishii M (2018). Cell-based chemical fingerprinting identifies telomeres and lamin A as modifiers of DNA damage response in cancer cells. Sci. Rep..

[41] Jacobsen PF, Jenkyn DJ, Papadimitriou JM (1985). Establishment of a human medulloblastoma cell line and its heterotransplantation into nude mice. J. Neuropathol. Exp. Neurol..

[42] Fu J, Shrivastava A, Shrivastava SK (2019). Triacetyl resveratrol upregulates miRNA-200 and suppresses the Shh pathway in pancreatic cancer: A potential therapeutic agent. Int. J. Oncol..

[43] Villunger A, O'Reilly LA, Holler N (2000). Fas ligand, Bcl-2, granulocyte colony-stimulating factor, and p38 mitogen-activated protein kinase: Regulators of distinct cell death and survival pathways in granulocytes. J. Exp. Med..

[44] Llaguno-Munive M, Romero-Piña M, Serrano-Bello J (2018). Mifepristone overcomes tumor resistance to temozolomide associated with DNA damage repair and apoptosis in an orthotopic model of glioblastoma. Cancers.

[45] Anstee NS, Bilardi RA, Ng AP (2019). Impact of elevated anti-apoptotic MCL-1 and BCL-2 on the development and treatment of MLL-AF9 AML in mice. Cell Death Differ..

[46] Seo BR, Min K-J, Woo SM (2017). Inhibition of cathepsin S induces mitochondrial ROS that sensitizes TRAIL-mediated apoptosis through p53-mediated downregulation of Bcl-2 and c-FLIP. Antioxid. Redox Signal..

[47] Canman CE, Gilmer TM, Coutts SB (1995). Growth factor modulation of p53-mediated growth arrest versus apoptosis. Genes Dev..

[48] Naeem A, Harish V, Coste S (2022). Regulation of chemosensitivity in human medulloblastoma cells by p53 and the PI3 kinase signaling pathway. Mol. Cancer Res..

[49] Northcott PA, Buchhalter I, Morrissy AS (2017). The whole-genome landscape of medulloblastoma subtypes. Nature.

[50] Miyake H, Tanabe K, Tanimura S (2020). Genetic deletion of vasohibin-2 exacerbates ischemia-reperfusion-induced acute kidney injury. Int. J. Mol. Sci..

[51] Tu M, Li H, Lv N (2017). Vasohibin 2 reduces chemosensitivity to gemcitabine in pancreatic cancer cells via Jun proto-oncogene dependent transactivation of ribonucleotide reductase regulatory subunit M2. Mol. Cancer.

[52] Rusert JM, Juarez EF, Brabetz S (2020). Functional precision medicine identifies new therapeutic candidates for medulloblastoma. Cancer Res..

[53] You H, Wei L, Kaminska B (2021). Emerging insights into origin and pathobiology of primary central nervous system lymphoma. Cancer Lett..

