# Supplementary figures and images for: A preliminary study on the mechanism of VASH2 in childhood medulloblastoma
Source: Sci Rep. 2023 Oct 11;13:17153. doi: 10.1038/s41598-023-42869-6 (PMC10567924; doi:10.1038/s41598-023-42869-6)

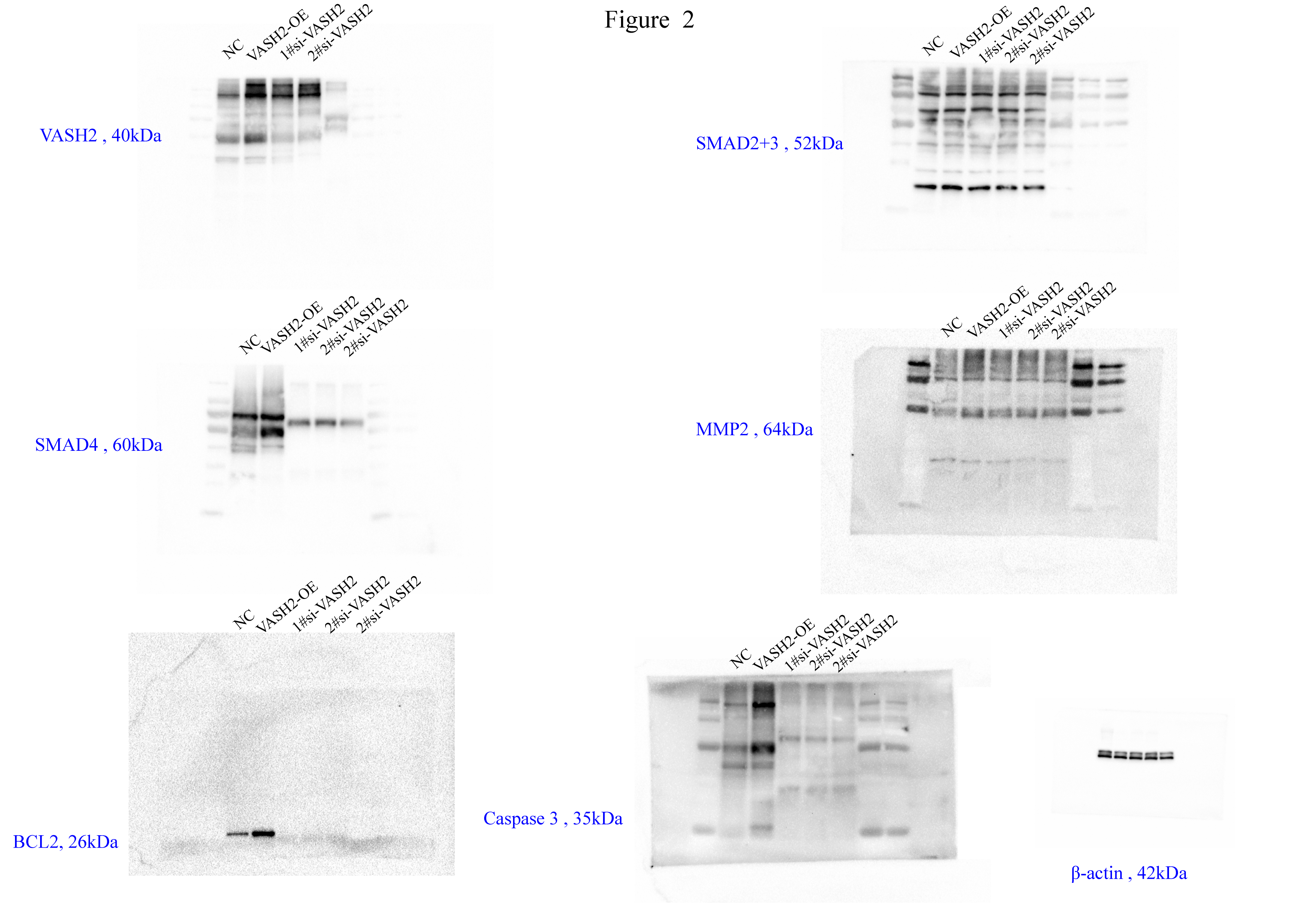

Supplement: Supplementary file 1 — Supplementary Information. [file 41598_2023_42869_MOESM1_ESM.tif]
